# Dielectric Sensors Based on Electromagnetic Energy Tunneling

**DOI:** 10.3390/s150407844

**Published:** 2015-03-31

**Authors:** Omar Siddiqui, Mani Kashanianfard, Omar Ramahi

**Affiliations:** 1 College of Engineering, Taibah University, University Road, Madinah, Saudi Arabia; E-Mail: ofsiddiqui@yahoo.com; 2 Department of Electrical Engineering and Computer Science, University of Michigan, Ann Arbor, MI 48109, USA; E-Mail: manikafa@yahoo.com; 3 Department of Electrical and Computer Engineering, University of Waterloo, 200 University Avenue West, Waterloo, ON N2L 3G1, Canada

**Keywords:** energy tunneling, dielectric sensors, metamaterials, metallic wire medium, waveguides

## Abstract

We show that metallic wires embedded in narrow waveguide bends and channels demonstrate resonance behavior at specific frequencies. The electromagnetic energy at these resonances tunnels through the narrow waveguide channels with almost no propagation losses. Under the tunneling behavior, high-intensity electromagnetic fields are produced in the vicinity of the metallic wires. These intense field resonances can be exploited to build highly sensitive dielectric sensors. The sensor operation is explained with the help of full-wave simulations. A practical setup consisting of a 3D waveguide bend is presented to experimentally observe the tunneling phenomenon. The tunneling frequency is predicted by determining the input impedance minima through a variational formula based on the Green function of a probe-excited parallel plate waveguide.

## 1. Introduction

The metallic-wire medium has been a source of interest among the electromagnetic community for the past several decades, primarily because of its similarities to the anisotropic plasmas. In his 1962 paper, Rotman studied the wire medium to model the wave propagation in plasmas by considering the analogous behaviors of their refractive indices (*i.e.*, dispersion) [1]. The wire medium gained popularity after it was applied, in combination with the split-ring resonators, in formulating the effective negative index medium [2]. The metallic wires, in this context, have been treated as an effective medium with negative permittivity in a certain range of frequencies, as given by the well-known relation:

(1) $$\epsilon_{r}=1-\frac{\omega^{2}}{\omega_{p}^{2}}$$

where *ω_p_* is the plasma frequency [3]. Many subsequent studies utilized their plasma-like dispersion characteristics to demonstrate interesting effects, such as resonance-cone focusing and epsilon-near-zero (ENZ) tunneling [4–9].

**Figure 1:**
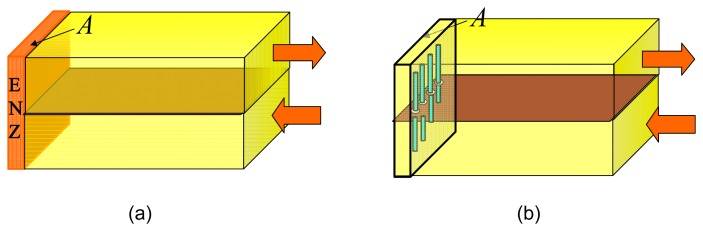
Different waveguide geometries that support electromagnetic energy tunneling. (**a**) Two short-circuited waveguides connected by a thin layer of epsilon-near-zero material; and (**b**) the two waveguides connected with a cylindrical wire structure.

This paper deals with the application of yet another exciting aspect of the wire medium dispersion characteristics, *i.e.*, the regime of the what has been referred to as “self-collimation”, “Bragg's refraction” or “canalization”. Such regimes are identified by diminishing group velocities along one of the structure's axes of symmetry and the flatness of the dispersion surfaces [10–13]. When such media are interfaced with free space, a strongly directive anomalous refraction is observed. The phenomenon has also been utilized to transport the image of an object with sub-wavelength details [12,13]. The electromagnetic energy propagation in such cases is confined to narrow channels and is determined by a particular relationship between the phase and group velocities [11]. If the impedance is correctly matched in these directions, full transmission of energy (in lossless cases) known as “tunneling” is possible [8]. The phenomenon has also been termed as “energy squeezing”, because of the possibility of transporting energy through sub-wave length channels [7,8]. Energy tunneling through narrow waveguide channels and bends filled with materials with ENZ electric permittivities have been explored in several studies [5–9]. One such arrangement depicted in Figure 1a is a 180° short-circuited bend in which two 3D rectangular waveguides are connected through a narrow channel filled with an ENZ material. In this waveguide configuration, the condition of full transmission is obtained by restricting the aperture A of the ENZ material to a small value [5]. Analogous energy tunneling effects have also been observed in waveguide bends loaded with wires, as shown in Figure 1b. In such a configuration, the metallic wires are separated by a periodicity of *T* and are directed parallel to the electric (E) field [14–16].

In the first two sections of this paper, we present a review of the energy tunneling phenomenon in waveguide bends through full-wave simulations using ANSYS^®^ HFSS^TM^. A dielectric sensing mechanism based on energy tunneling is demonstrated. Section 3 provides experimental evidence of the phenomenon in a 3D rectangular waveguide. In Section 4, we expand our previous works [14–16] by providing an analytical formulation of the wire-based waveguide tunneling. The developed analytical method explains the underlying tunneling mechanism and predicts the transmission frequency of the resonant wire. The proposed method is based on the Green function formulation of impedance and is inspired by a similar problem of probe antennas when they excite rectangular waveguides. Note that the ENZ-based tunneling exploits the effective medium characteristics when the dispersion surfaces become hyperbolic. The wire-based tunneling, on the other hand, is observed in the long wavelength dispersion region when the unit cell is comparable to the wavelength. The tunneling concept proposed in this paper has the flexibility of tuning the cavities to a desired frequency without any waveguide modifications. Particularly, more resonances can be added to operate the waveguides at multiple tunneling frequencies. Furthermore, the tunneling effects in the ENZ-based cavities at microwave frequencies are obtained by using metamaterial-based periodic structures that are inherently dispersive and possess large loss tangents, especially in the frequency ranges where the permittivities are near zero.

## 2. The Energy Tunneling Sensor

### 2.1. The Tunneling Mechanism

**Figure 2:**
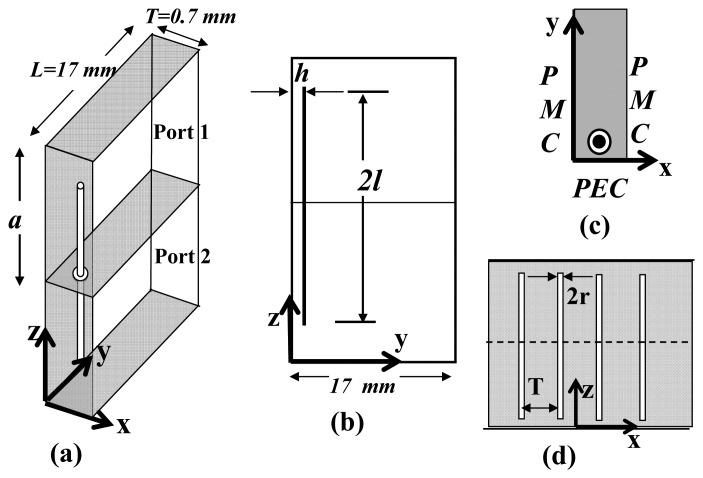
**(a)** Unit cell of the parallel-plate waveguide configuration that supports energy tunneling. The shaded walls are the perfectly electric conductors (PECs) and the un-shaded walls are perfectly magnetic conductors (PMCs); (**b**) side view and (**c**) top view showing the boundary conditions; (**d**) the infinitely extended x-y plane. Waveguide dimensions are given by *a* = 3.56 mm, *r* = 0.04 mm, *R* = 0.12 mm and *h* = 0.178 mm. (The picture is not drawn to the scale.)

**Figure 3:**
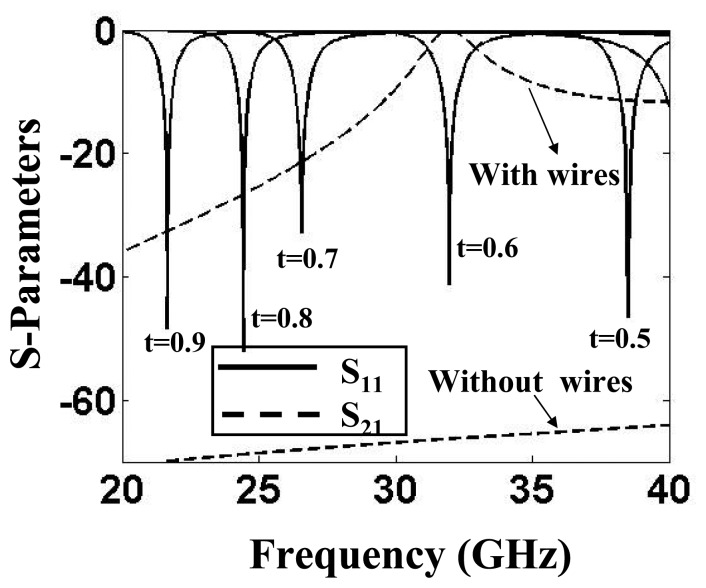
Reflection (solid lines) and transmission (dashed lines) coefficients for different values of the wire length to waveguide height ratio (*t* = 2*l*/2*a*).

The phenomenon of energy-tunneling is supported by the waveguide configurations in which the electric fields are excited parallel to the wires [14]. Examples include strip line, parallel-plate waveguide and microstrip transmission lines. Consider an energy tunneling setup of two short-circuited parallel-plate waveguides connected through a narrow aperture. This is a two-dimensional waveguide configuration and can be represented in a unit-cell domain shown in Figure 2. The unit-cell simulation method is relatively fast and efficient for the analysis of structures that are electrically very large. Moreover, it is simple to configure in electromagnetic simulation tools, such as ANSYS® HFSS^TM^. As shown, each waveguide that constitutes the bend is backed by a perfectly conducting wall and has a height of *a* and a width of *T*. The two waveguides are connected through an aperture whose radius is *R*, which contains a metallic wire of radius *r*. With two perfectly-magnetic side walls, as indicated in the figure, the unit cell behaves as an infinitely-extended structure along the x-axis. The TEM mode in one waveguide is excited through the ANSYS^®^ HFSS^TM^ wave port. Assuming zero loss, the resulting magnitude of the reflection coefficients (|*S*_11_|) for different values of wire length to waveguide height ratio (*t*) are plotted in Figure 3. Evidently, the points of |*S*_11_| minima corresponds to the tunneling frequency. The shift in the resonance shows that the tunneling frequency can be tuned by changing the length of the wires. To show the no-loss energy transfer at the tunneling frequency, the magnitude of the transmission coefficient |*S*_21_| for one case (*t* = 0.6) is also depicted. The |*S*_21_| plot shows that under lossless conditions, a complete power transfer results from one waveguide to the other at around 31.96 GHz. Note that without the wires, no transmission is possible through the apertures. A distinctive feature of the wire-based tunneling is the very high electric field concentration around the wire tips as a result of tunneling wave propagation through a channel that only comprises a very thin wire. Therefore, in comparison with a resonant waveguide, the wire-based energy tunneling offers very high sensitivity to dielectric samples placed around the locations of concentrated energy.

**Figure 4:**
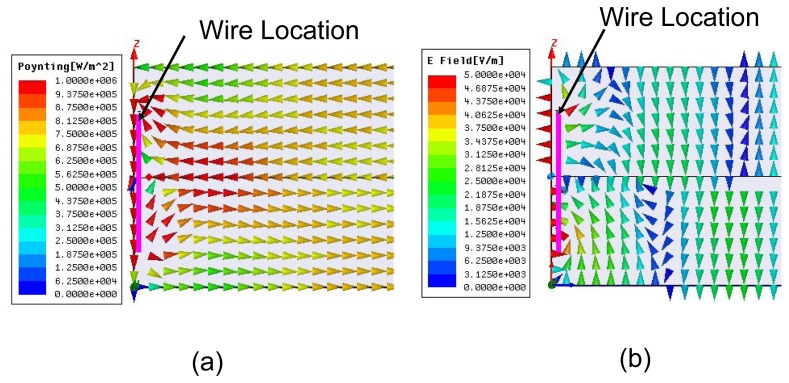
**(a)** Poynting vector ((power flow) plot and (**b**) electric field pattern on the magnetic wall (y-z plane) of the parallel plate waveguide unit cell at 31.96 GHz for the case in which the wire-length to waveguide-height ratio *t* = 0.6.

The properties of the wire-based tunneling can be further investigated by plotting the real power vector diagram and electric field patterns (Figure 4) evaluated at the perfect magnetic conductor (PMC) walls of the unit cell at 31.96 GHz for the *t* = 0.6 case. The power diagram (Figure 4a) clearly corroborates the fact that the propagation of energy is confined in the narrow channel between the wire and the conducting waveguide wall. The electric field pattern shows that the waveguide TEM electric fields are coupled to the wire's resonant mode at the tunneling frequency. As expected, a high concentration of the electric field, oriented perpendicular to the wire, is detected on and in the vicinity of the wire. Away from the wire, the vertical fields suggest that the dominant mode of propagation is TEM, which intrinsically flows in a parallel-plate waveguide. The electric field pattern also sheds light on the differences between the tunneling mechanisms in the wire-loaded waveguides and the analogous effects in the ENZ-based waveguides. The ENZ tunneling is characterized by uniform and enhanced electric field distributions throughout the channel, resulting in very small phase variations [6]. On the other hand, the tunneling in wire-loaded waveguides is governed by resonances that are characterized by large phase changes and non-uniform electric (and magnetic) field distributions. As shown in Figure 4b, the strong non-uniform electric field experiences a 180° phase variation as it propagates through the channel at the 31.96-GHz resonance.

### 2.2. Operation of a Dielectric Sensor

**Figure 5:**
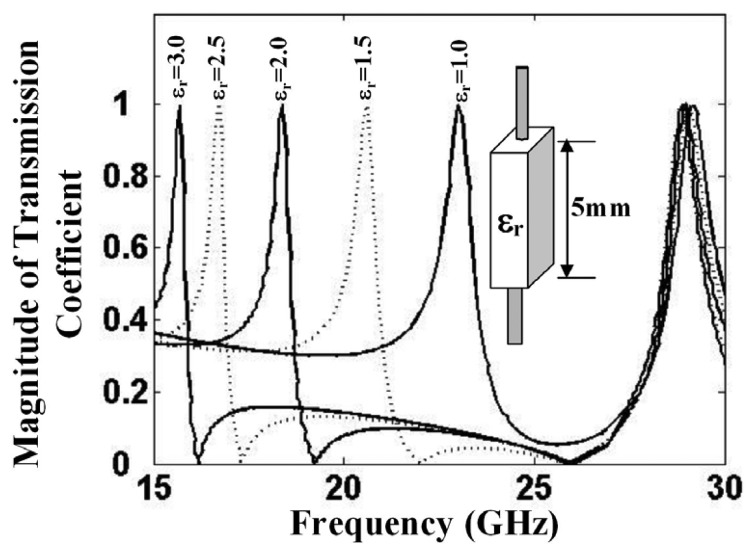
Transmission coefficients for wire-loaded waveguide cavity with the wire covered with different values of permittivities (reproduced from [16] with the authors' permission).

**Figure 6:**
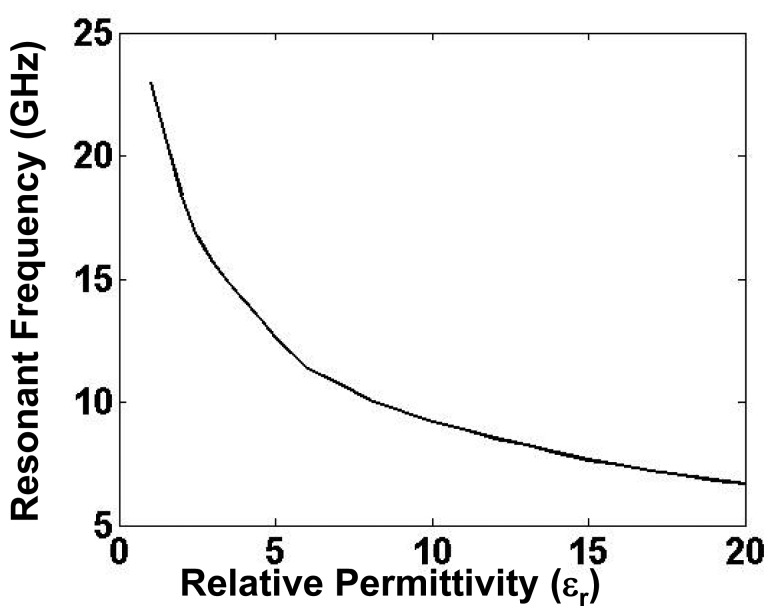
The characteristic curve of the detector showing the variation of the resonant frequency with the dielectric permittivity.

As depicted in Figure 3, the resonant frequency of the tunneling waveguide cavity shifts to lower values with the increase in wire length. The effective length of the wire can be also increased by immersing it into a dielectric of higher relative permittivity. Conversely, a dielectric sensor can be fabricated that can determine the permittivity values by detecting the resonance of the tunneling waveguide. A practical dielectric sensor is presented in [16]. The sensing behavior can be observed in the transmission curves shown in Figure 5 for a tunneling setup that was designed to resonate at 23 GHz in the absence of a dielectric. When a 5-mm dielectric sample was inserted, the transmission peak shifted to lower resonances. As depicted in Figure 5, the sensor indeed supports a tunneling mode that is highly sensitive, especially in the lower permittivity range. For example, a small permittivity shift from *ε_r_* = 1 to *ε_r_* = 1.5 results in a large resonance shift of −2.4 GHz. Practically, a characteristic curve of the detector, similar to the one shown in Figure 6, can be obtained at the time of fabrication. Note that when the permittivity attains higher values, the characteristic curve approaches saturation imposed by the law of charge conservation. In Figure 5, a second resonance peak is also observed at around 29 GHz for all of the dielectric values. This peak is due to the well-known Fabry–Perot resonance that builds up in a closed cavity. The Fabry–Perot resonances depend on the channel dimensions and are independent of the wires' length; hence, they are observed at a constant frequency.

## 3. An Energy Tunneling Experiment Using 3D Waveguides

**Figure 7:**
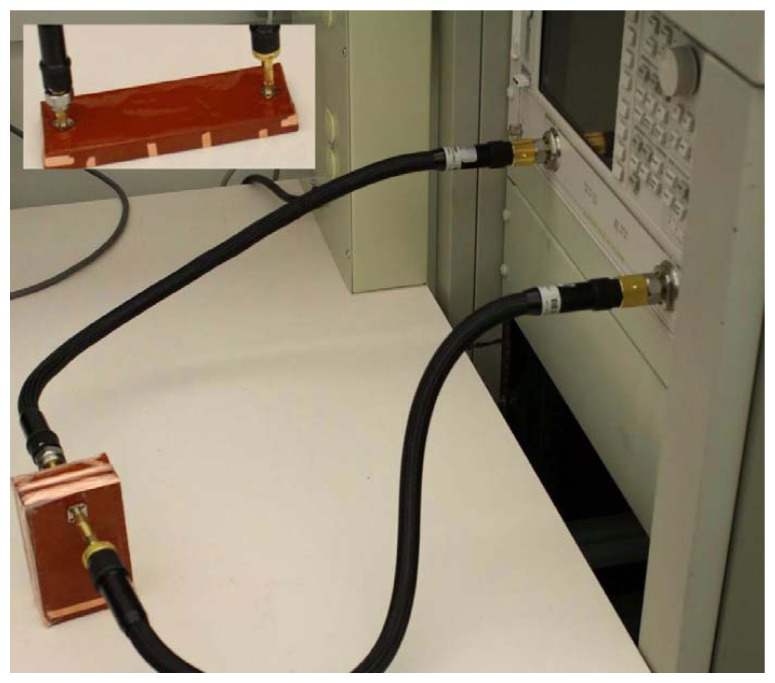
The experimental setup to verify energy tunneling in a wire-loaded waveguide bend. The inset shows the control waveguide.

**Figure 8:**
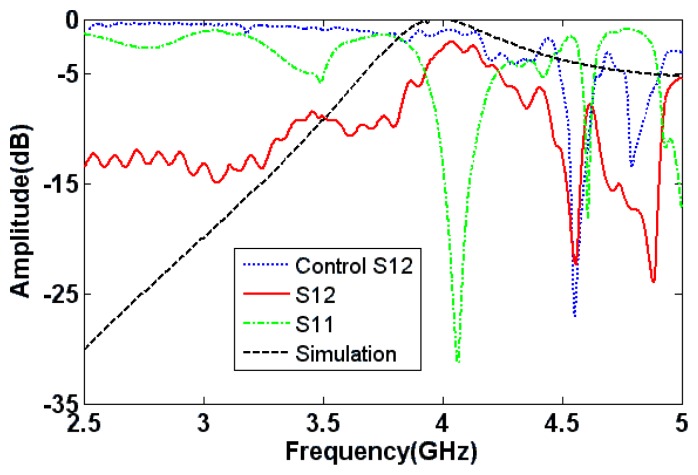
Experimental S-parameters for the 3D waveguide bend, the control waveguide. The simulation transmission coefficient (*S*_21_) is also shown for comparison.

The 3D waveguide configuration, shown in Figure 1, can be used to demonstrate the concept of wire-based energy tunneling. A practical waveguide measurement setup is depicted in Figure 7. The waveguide is fabricated by wrapping conducting copper sheets over a slab made up of Plexiglas (*ε_r_* = 2.57). The thickness of the waveguide is 17 mm, and the width of the waveguide is 63.5 mm. With these dimensions, the waveguide has a cutoff frequency of 1.48 GHz. The steel wires that are placed at the end of the waveguide measure 20 mm in length (*l* = 10 mm) and 3.2 mm in diameter. The diameter of the aperture is 2*R* = 9.5 mm, and the wires are separated by a distance of *T* = 12.7 mm. A control waveguide, shown in the inset of Figure 7, was also fabricated to characterize the losses in the experimental setup. The waveguide is fed with an SMA connector connected to an Agilent HP8722ES vector network analyzer through a coaxial cable. The experimental S-parameters of the waveguide bend and the control waveguide are shown in Figure 8. ANSYS^®^ HFSS^TM^ simulations are also included in the plot for comparison. The tunneling effect is observed at about 4 GHz. The control waveguide measurement shows about 1.5 dB loss at the transmission frequency. These losses are mainly caused by the impedance mismatches at the SMA connectors, the dielectric and metallic losses in the system and the fabrication imperfections. An additional 1 dB loss in the 180° bend is partly due to the metallic conductors. The simulation, however, shows negligible losses when the conductor losses in the wires and waveguide dielectric losses are considered. Additional resonances observed in the experimental results are due to the cavity effect.

## 4. Analytical Formulation of the Resonance Mechanism

**Figure 9:**
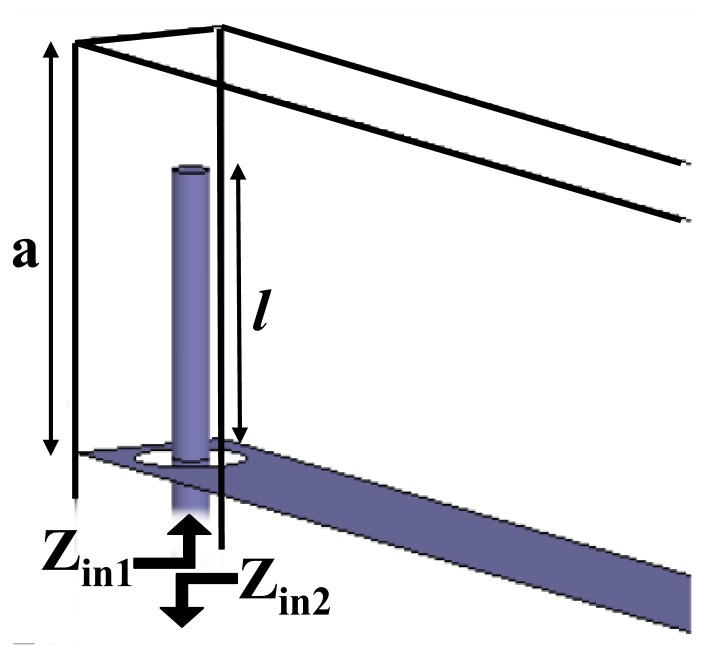
Definition of the input probe impedance.

The tunneling frequency of the wire-loaded parallel-plate waveguide can be calculated analytically by considering the antenna probe analogy that excites a rectangular waveguide [17]. Looking at Figure 2, the configuration resembles two probes that are connected back to back exciting two waveguides. The input impedances *Z_in_*_1_ and *Z_in_*_2_ can thus be defined at the entrances of two interconnected parallel-plate waveguides, as depicted in Figure 9. At the tunneling frequency, the energy is coupled from one waveguide to the other when the two probes operate at the impedance-matched condition. Numerically, this condition is obtained when the input impedances of the two probes are complex conjugates of each other. Moreover, because of the symmetry of the problem, *Z_in_*_,1_ = *Z_in_*_,2_, which can only be possible if the imaginary part of the input impedance of each probe is zero. The input impedance can be written in terms of the current density and the dyadic Green function in the form of the following variational formula [17]:

(2) $$Z_{in}=\frac{1}{I_{in}^{2}}\int_{S_{o}}\int_{S_{o}}J\left(r\right)\bar{G}\left(r|r^{\prime }\right)J\left(r\right)dSdS^{\prime }$$

where *S_o_* is the surface of the probe inside the waveguide. Assuming a y-directed uniform angular current density *J(r)* = *I(y)/2r*, the Green function is given by the following expression [17]:

(3) $$G_{yy}=\frac{jZ_{o}}{aTk_{o}}\sum_{n=1}^{\infty }\sum_{m=1}^{\infty }\frac{\epsilon_{om}\epsilon_{on}k_{m}^{2}}{\Gamma_{nm}}\cdot \cos\frac{n\pi x}{T}\cdot \cos\frac{n\pi x^{\prime }}{T}\cdot \cos\frac{m\pi y}{T}\cdot \cos\frac{m\pi y^{\prime }}{T}\cdot e^{-\Gamma_{nm}\left(z_{>}+h\right)}\cdot \text{sinh}\Gamma_{nm}\left(z_{>}+h\right)$$

where *Z_o_* and *k_o_* are the intrinsic impedance and wavenumber of the medium that fills the waveguide, *ε_on_* is the Neumann factor, which is equal to zero for *n* = 0 and equals two otherwise. $k_{m}^{2}={\left(m\pi /a\right)}^{2}-k_{o}^{2}$ and $\Gamma_{nm}^{2}={\left(n\pi /T\right)}^{2}+k_{m}^{2}$ . *z*_>_ is the larger of *z* and *z′* and *z*_<_ is the smaller of the two. The last two factors in Equation (3) can be written as:

(4) $$\cos\frac{m\pi y^{\prime }}{T}\cdot e^{-\Gamma_{nm}\left(z_{>}+h\right)}\cdot \text{sinh}\Gamma_{nm}\left(z_{>}+h\right)=\frac{1}{2}\left\{e^{-\Gamma_{nm}\left|z-z^{\prime }\right|}-e^{-2\Gamma_{nm}h-\left(z+z^{\prime }\right)\Gamma_{nm}}\right\}$$

Substituting Equations (3) and (4) in Equation (2) yields,

(5) $$Z_{in}=\frac{1}{{\left[I\left(0\right)\right]}^{2}}\frac{jZ_{o}}{ak_{o}}\sum_{m=0}^{\infty }\left\{\left[\frac{\epsilon_{\text{om}}k_{m}^{2}}{4\pi^{2}}\cdot I_{1}-\sum_{n=0}^{\infty }\frac{\epsilon_{\text{om}}\epsilon_{\text{on}}k_{m}^{2}}{4\pi^{2}\Gamma_{nm}}\cdot I_{2}\right]\cdot I_{3}\cdot I_{4}\right\}$$

where the integrals *I*_1_ − *I*_4_ are defined by:

(6) $$I_{1}=\int_{0}^{2\pi }\int_{0}^{2\pi }\left(\sum_{m=0}^{\infty }\frac{\epsilon_{om}}{2}\frac{\cos\frac{n\pi x}{T}\cos\frac{n\pi x^{\prime }}{T}}{\Gamma_{nm}T}e^{-\Gamma_{nm}\left|z-z^{\prime }\right|}\right)d\phi d\phi^{\prime }$$

(7) $$I_{2}=\int_{0}^{2\pi }\int_{0}^{2\pi }\left(\cos\frac{n\pi x}{T}\cos\frac{n\pi x^{\prime }}{T}e^{-2\Gamma_{nm}h-\left(z+z^{\prime }\right)\Gamma_{nm}}\right)d\phi d\phi^{\prime }$$

(8) $$I_{3}=\int_{0}^{l}\cos\frac{m\pi y}{a}I\left(y\right)dy$$

(9) $$I_{4}=\int_{0}^{l}\cos\frac{m\pi y^{\prime }}{a}I\left(y^{\prime }\right)dy^{\prime }$$

Following a similar procedure that is outlined in [17], which involves writing the Equation (5) in terms of cylindrical Bessel and Hankel functions and integrating over *ϕ* and *ϕ′*, we can arrive at the following simpler expression for the input impedance:

(10) $$Z_{in}=\frac{1}{{\left[I\left(0\right)\right]}^{2}}\sum_{m=0}^{\infty }g_{m}\cdot I_{3}\cdot I_{4}$$

where *g_m_* for indexes *m* > 0 can be calculated as:

(11) $$g_{m}=\frac{jZ_{o}}{ak_{o}}\frac{k_{m}^{2}}{\pi }\left(I_{o}\left(k_{m}r\right)K_{o}\left(k_{m}r\right)+\sum_{n=1}^{\infty }2K_{o}\left(k_{m}nT\right)\right)-\frac{jZ_{o}}{ak_{o}}\left(1+\frac{k_{m}^{2}r^{2}}{2}\right)\left(\frac{k_{m}}{T}e^{-2k_{m}h}+2k_{m}^{2}\sum_{n=2,4,..}^{\infty }\frac{e^{-2\Gamma_{nm}h}}{\Gamma_{nm}T}\right)$$

where *I_o_* and *K_o_* are the modified Bessel functions of the first and second kinds, respectively. *g_o_* is given by:

(12) $$\begin{array}{l} g_{o}=-\frac{jZ_{o}k_{o}}{a}\left[\frac{-j\left(J_{o}\left(k_{o}r\right)-1\right)H_{o}^{2}\left(k_{o}r\right)}{4}+\frac{e^{-jk_{o}r}}{2jk_{o}r}-\frac{\ln\left(1-e^{-2\pi r/a}\right)}{2\pi }\right]\\ -\frac{jZ_{o}k_{o}}{a}\overset{\infty }{\underset{n=2,4,..}{\text{∑}}}\left(\frac{e^{-\Gamma_{no}r}}{\Gamma_{no}T}-\frac{e^{-n\pi r/a}}{n\pi }\right)+\frac{jZ_{o}k_{o}}{a}\left(1-\frac{k_{o}^{2}r^{2}}{2}\right)\left(\frac{e^{-2jk_{o}h}}{2jTk_{o}}+\overset{\infty }{\underset{n=2,4,..}{\text{∑}}}\frac{e^{-2\Gamma_{no}h}}{\Gamma_{no}T}\right) \end{array}$$

where *J_o_* and *H_o_* are the Bessel and Hankel functions, respectively In order to find the best choice of currents on the wires, we use a set of basis functions *ψ_ν_*(*y*) to form *I*(*y*):

(13) $$I\left(y\right)=\sum_{\nu =1}^{q}I_{\nu }\psi_{\nu }\left(y\right)$$

If the current basis function *ψ_ν_*(*y*) is normalized, such that *ψ_ν_*(0) = 1, then:

(14) $$I\left(y\right)=\sum_{\nu =1}^{p}I_{\nu }$$

The impedance Equation (10) can the be written in the following matrix form:

(15) $${\text{Z}}_{in}=\frac{{\text{I}}^{\text{T}}\text{GI}}{{\text{I}}^{\text{T}}\text{NI}}$$

where,

(16) $$\text{I}={\left[I_{1}I_{2}\ldots I_{q}\right]}^{T}$$

(17) $${\left[G\right]}_{ij}=\sum_{m=0}^{\infty }g_{m}P_{i}^{\left(m\right)}P_{j}^{\left(m\right)}$$

and **N** is the unitary matrix. The $P_{\nu }^{\left(m\right)}$ is defined as the following integral:

(18) $$P_{\nu }^{\left(m\right)}=\int_{0}^{l}\cos\frac{m\pi y}{a}\psi_{\nu }\left(y\right)dy$$

Since the best approximation of the input impedance is obtained when Equation (15) is stationary, or in other words:

(19) $$\frac{\text{d}}{\text{dI}}Z_{in}=-2\frac{\left({\text{I}}^{\text{T}}\text{GI}\right)\left({\text{I}}^{\text{T}}\text{N}\right)-\left({\text{I}}^{\text{T}}\text{NI}\right)\left({\text{I}}^{T}\text{G}\right)}{{\left({\text{I}}^{T}\text{NI}\right)}^{2}}=0$$

To solve Equation (19) numerically, the following four basis current functions are selected:

(20) $$\begin{array}{l} \psi_{1}=\frac{{\text{sink}}_{o}\left(l-y\right)}{\sin k_{o}l},\psi_{2}=\frac{1-{\text{cosk}}_{o}\left(l-y\right)}{1-{\text{cosk}}_{o}l}\\ \psi_{3}=\cos\frac{\pi y}{2l},\psi_{4}=\cos\frac{3\pi y}{2l} \end{array}$$

**Figure 10:**
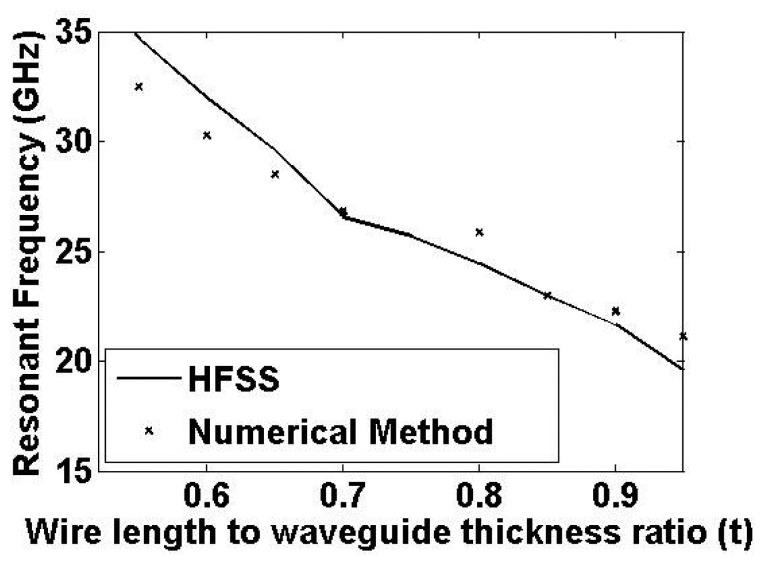
Plot of the first two principal components, which contributed more than 70% of the variance.

The analytical method determines the tunneling frequency by searching the frequency at which the input impedance Equation (15) has a vanishing imaginary part. The results for different values of wire lengths in the air-filled waveguide of Figure 2 are displayed in Figure 10. Along with the numerical results, the resonant frequencies obtained by the ANSYS^®^ HFSS^TM^ are also shown. The difference between the two results is about 6% in the worst case. We note that the numerical results based on the impedance matching of interconnected probes are approximate, as numerical approximations were used; therefore, we expect the full-wave simulation to give a more accurate prediction of the resonant frequencies. The time required to generate the theoretical results is much less than the time required to run the simulations. Therefore, the numerical solution should be used as an initial step in the design procedure. The final design values can then be fine-tuned utilizing the full-wave simulations. When the waveguide is filled with a dielectric instead of air, the resonant frequency shifts towards a lower value. For instance, for *t* = 0.9, the numerical method predicts the new resonance at 20.52 GHz when the waveguide dielectric constant is changed to 1.3.

## 5. Conclusions

We studied theoretically and experimentally the the frequency-dependent tunneling of electromagnetic energy as it propagates through narrow bends and channels loaded with resonant wires. In particular, a 180° waveguide bend loaded with metallic wires was designed to tunnel electromagnetic energy with full transmission under ideal conditions. The tunneling is characterized by intense electric field distributions and a highly selective frequency response, which can be utilized in the determination of the dielectric properties of the media. The frequency response of such wire-loaded cavities primarily depends on the lengths of the resonant wires and, therefore, can be tailored to any desired range of frequencies. The wires in the waveguide configuration were analyzed by writing the variational impedance formula in a way that was similar to that of a probe-excited waveguide. A maximum difference of 6% was observed when the resonant frequencies obtained from the analytical method were compared to the ANSYS^®^ HFSS^TM^ simulations. The tunneling in the wire-based waveguides is characterized by non-uniform spatial field distributions and large phase changes that resemble a Fabry–Perot resonance. Due to the ease of fabrication, flexibility in tuning without waveguide modifications and scalability to any desired range of frequencies, the proposed configuration of wire-loaded waveguide bends can also find wide applications in microwave and millimeter wave bands.
